# Dual-Functional CeO_2_ Nanozyme-Based Fluorescent Sensing Platform for Chiral Recognition of Arginine and “On-Off-On” Detection of *p*-Nitrophenol and Alkaline Phosphatase

**DOI:** 10.3390/molecules31122003

**Published:** 2026-06-08

**Authors:** Hui-Ling Chen, Jing-Jing Dai, Hua Chen, Guo-Ying Chen, Feng-Qing Yang

**Affiliations:** School of Chemistry and Chemical Engineering, Chongqing University, Chongqing 401331, China; 202518021106t@stu.cqu.edu.cn (H.-L.C.); 202318021129t@stu.cqu.edu.cn (J.-J.D.); chenhuacqu@cqu.edu.cn (H.C.)

**Keywords:** CeO_2_ nanoflower, tandem enzymes, fluorescent switch, arginine chiral recognition, alkaline phosphatase detection

## Abstract

Nanomaterials with multiple enzyme-like activities offer significant opportunity for constructing multifunctional sensing methods. In this work, a hydrangea flower-like cerium dioxide nanomaterial (CeO_2_ NF) with both peroxidase (POD)- and hydrolase-like activities, which was surface-modified by polyvinylpyrrolidone (PVP) in situ, was prepared through an oil bath method. Based on the POD-like activity of CeO_2_ NFs, an “on-off” fluorescence method was established for chiral recognition of arginine (Arg) enantiomers. Meanwhile, utilizing the hydrolase-like activity of CeO_2_ NFs and their synergistic interaction with alkaline phosphatase (ALP), an “on-off-on” fluorescence method was developed for the detection of *p*-nitrophenol (*p*-NP) and ALP. The sensor demonstrated excellent chiral selectivity for Arg enantiomers, with a high enantiomeric factor (ef) of up to 2.48, allowing for the quantitative detection of L-Arg in the range of 770–940 μM, with a limit of detection (LOD) of 26.00 μM. Furthermore, it exhibited high sensitivity for *p*-NP and ALP detection, with linear ranges of 10.0–84.3 μM and 300–2000 mU/mL, and LODs of 7.07 μM and 200 mU/mL, respectively. Through an enzyme kinetic analysis, fluorescence lifetime measurement, zeta potential analysis, and density functional theory (DFT) calculations, the underlying catalytic and chiral recognition mechanisms were proposed. Finally, the method was validated through the accurate detection of L-Arg, *p*-NP, and ALP in real samples (rabbit plasma, food-grade amino acid, and water samples).

## 1. Introduction

Amino acids (AAs) are the fundamental building blocks of proteins and play vital roles in daily biological processes. Aside from glycine, all common AAs exist as stereoisomers [1]. Due to the stereochemical configuration and the stereoselective recognition mechanisms of biological macromolecular receptors, AA enantiomers exhibit significant functional differences. Typically, L-AAs display positive bioactivity in organisms, whereas D-AAs are either inactive or may exert adverse effects. These distinct properties make the study and application of these functional disparities of great significance across various fields, including pharmaceuticals [2], life sciences [3], food safety [4], and environmental protection [5]. For example, L-Tryptophan (L-Trp), an essential AAs, serves not only as a fundamental substrate for protein biosynthesis but also as a precursor for serotonin. Serotonin plays a critical role in the central nervous system, participating in mood regulation, sleep cycle control, and nociception, with its synthesis dependent on the transport of L-Trp across the blood–brain barrier and the specific catalytic action of hydroxylase enzymes [6]. Conversely, D-Tryptophan (D-Trp), due to its stereochemical configuration, cannot be directly utilized by mammalian metabolic systems. Instead, it can be converted into D-Alanine (D-Ala) by peptidoglycan synthesis pathways in bacteria, becoming a structural component of peptide antibiotics, such as vancomycin [7]. This strict stereochemistry–function relationship exemplifies the precise regulatory role of chiral molecules in biological processes. Therefore, the separation and selective recognition of enantiomers are crucial for medicine, the environment, and life sciences. Alkaline phosphatase (ALP), known for its high catalytic efficiency and broad substrate specificity, plays a vital role in biological metabolism [8]. ALP can catalyze the dephosphorylation of amino acid phosphates, such as tyrosine phosphate. Its activity depends on the specific amino acid residues and metal ions at the active site, essential for amino acid metabolism and cell regulation [9]. Furthermore, it is widely employed as an enzymatic label in immunoassays and as a biomarker for bone and hepatobiliary diseases diagnosis [10]. Consequently, the development of highly sensitive methods for measuring ALP activity is of paramount importance for diagnostic and analytical applications.

The commonly employed techniques for AA enantiomer recognition and ALP activity detection are high-performance liquid chromatography [11,12], gas chromatography [13], capillary electrophoresis [14,15,16], circular dichroism spectroscopy [17,18,19], electrochemical methods [20,21], and fluorescence spectroscopy [22,23,24]. These analytical approaches often require expensive instrumentation and are limited in their widespread application in practical analysis due to their operational complexity and labor-intensive sample preparation processes [25]. Compared to these analytical methods, the fluorescence technique offers several advantages, such as high sensitivity, rapid analysis, and quick response, making it suitable for the identification of chiral AAs and quantitative determination of ALP activity in various samples [23]. Natural enzymes usually have binding sites with complementary shapes, charges and hydrophilic/hydrophobic properties to those of their substrates, enabling specific recognition [26]. Consequently, they can selectively identify or catalyze the transformation of highly similar substrate molecules, finding application in AA chiral recognition and the construction of fluorescent sensors for ALP detection [27]. Nanomaterials with enzyme-like activities (nanozymes) offer unique advantages over natural enzymes, including low cost, adjustable activity, and high stability under harsh conditions, thereby overcoming the limitations of high cost, operational instability, and difficulties of large-scale production associated with natural enzymes [28]. Therefore, they are finding broader application in AA enantiomer recognition and ALP activity detection. For example, Liu et al. [29] successfully constructed a bimetallic nanozyme composite material of LW@AuNPs-LWLH@CuNPs, which exhibited remarkable peroxidase (POD)-like activity. Trp enantiomers can adsorb onto LW@AuNPs-LWLH@CuNPs via hydrogen bonding, preventing substrate access to the active sites and further inhibiting product generation. Due to the stronger hydrogen bonding between L-Trp and the chiral ligand, the nanozyme can selectively recognize L-Trp with high stereospecificity (the chiral selectivity factor is 4.16). Chen et al. [30] successfully constructed the composite nanomaterial CuO NPs@ZIF-8, and applied it to an ALP activity analysis. This material possesses dual catalytic properties, with hydrolase- and oxidase (OXD)-like activities. Combining fluorescence and colorimetric (UV-Vis) analyses, the rapid and highly sensitive detection of ALP and glucose oxidase (GOX) was realized across a broad linear range (fluorescence for GOX: 0.86–1.23 × 10^5^ mU/mL; UV-Vis for GOX: 0.081–1.62 × 10^5^ mU/mL; fluorescence for ALP: 0.042–1.20 × 10^4^ mU/mL; UV-Vis for ALP: 0.0046–1.23 × 10^4^ mU/mL) with low LOQs (fluorescence for GOX: 0.86 mU/mL; UV-Vis for GOX: 0.081 mU/mL; fluorescence for ALP: 0.042 mU/mL; UV-Vis for ALP: 0.0046 mU/mL).

Cerium dioxide (CeO_2_) nanozymes, owing to their unique Ce^3+^/Ce^4+^ redox cycle and abundant surface oxygen vacancies, exhibit a variety of enzymatic-like activities, including POD-, OXD-, catalase- (CAT-), and superoxide dismutase(SOD-)-like activities, and have been widely applied in fields such as analytical chemistry, food science, and biomedicine [31,32]. For example, Fu et al. [33] employed a sonochemical doping strategy to co-modify the surface of nano-ceria with copper ions and 2-propylimidazole, preparing a novel CeO_2_@Cu-PrIm nanozyme. This material exhibited excellent OXD-like activity and was successfully used for the analysis of total antioxidant capacity (TAC) in fruits, with a detection limit as low as 1.26 μM. On the other hand, Lee et al. [34] reported a zinc-doped mesoporous ceria (Zn-m-ceria) engineered to possess two distinct functional sites—one for adsorbing organophosphates (OPs) and water, and the other acting as a general base catalyst—demonstrating significant organophosphate hydrolase (OPH)-like activity and enabling the sensitive detection of methyl paraoxon. It is notable that reports on the use of ceria for chiral recognition remain limited and typically require complex surface modifications. For example, Sun et al. [35] grafted D-/L-Phenylalanine onto the surface of CeO_2_ nanoparticles to construct a stereoselective nanozyme, utilizing its inherent OXD-like activity to achieve diastereoselective oxidation of dopamine (DOPA) enantiomers. Furthermore, integration of multiple enzymatic-like activities on a single CeO_2_ platform remains an under-explored topic.

**Figure 1:**
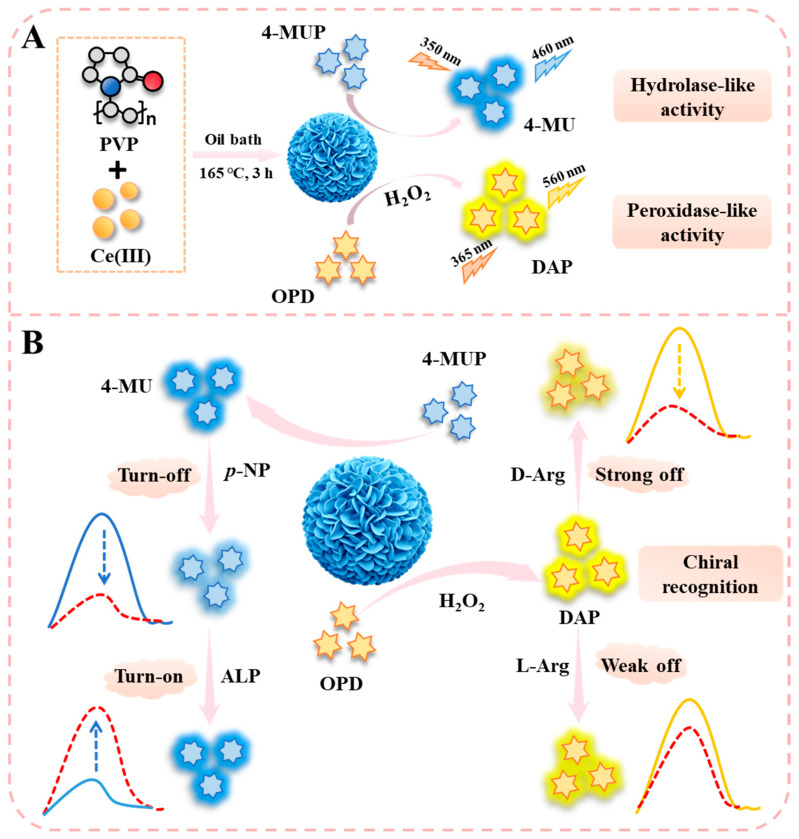
Schematic diagrams of the synthesis of CeO_2_ NFs with POD-/hydrolase-like activities (**A**), and their application to enantioselective recognition of Arg enantiomers and detection of *p*-NP and ALP (**B**).

In this study, turning “on-off” and “on-off-on” fluorescence sensors are engineered for selective recognition of Arg enantiomers and quantitative analysis of ALP activity, based on the outstanding multiple enzyme-like activity of CeO_2_ NFs (POD-/hydrolase-like activities) (Figure 1), which were surface-modified by polyvinylpyrrolidone (PVP) in situ during their synthesis. Leveraging the POD-like activity of CeO_2_ NFs, o-phenylenediamine (OPD) can be catalytically oxidized in the presence of H_2_O_2_ to yield 2,3-diaminophenazine (DAP), which exhibits yellow fluorescence with an emission peak at 560 nm, thereby switching the fluorescence signal to an “on” state. Upon the introduction of L/D-Arg enantiomers, the fluorescence intensity of the system is quenched, switching the signal to an “off” state. This distinct fluorescence response (“on-off’’) forms the basis for the chiral recognition and quantitative determination of Arg enantiomers. Capitalizing on the hydrolase-like activity of CeO_2_ NFs, the substrate 4-methylumbelliferyl phosphate (4-MUP) can be catalytically hydrolyzed to 4-methylumbelliferone (4-MU), which produces a blue fluorescence emission at 460 nm, thereby initiating the system in the fluorescence “on” state. The introduction of *p*-nitrophenol (*p*-NP) quenches this fluorescence, switching it “off”. Subsequently, ALP is added to the system to hydrolyze the remaining 4-MUP, thereby restoring the fluorescent signal to the “on” state. This ingeniously designed fluorescence “on-off-on” enables the highly sensitive detection of both *p*-NP and ALP. The effects of key synthetic parameters, including ligand concentrations and synthesis time, on the morphology, structure, and enzymatic activity of the CeO_2_ NFs are systematically investigated. The corresponding multi-enzyme catalytic kinetics and underlying reaction mechanisms are further elucidated. Finally, the developed methods are successfully applied to the chiral recognition of Arg enantiomers in complex samples, and for the sensitive detection of ALP and *p*-NP.

## 2. Results and Discussion

### 2.1. Characterization of CeO_2_ NFs

**Figure 2:**
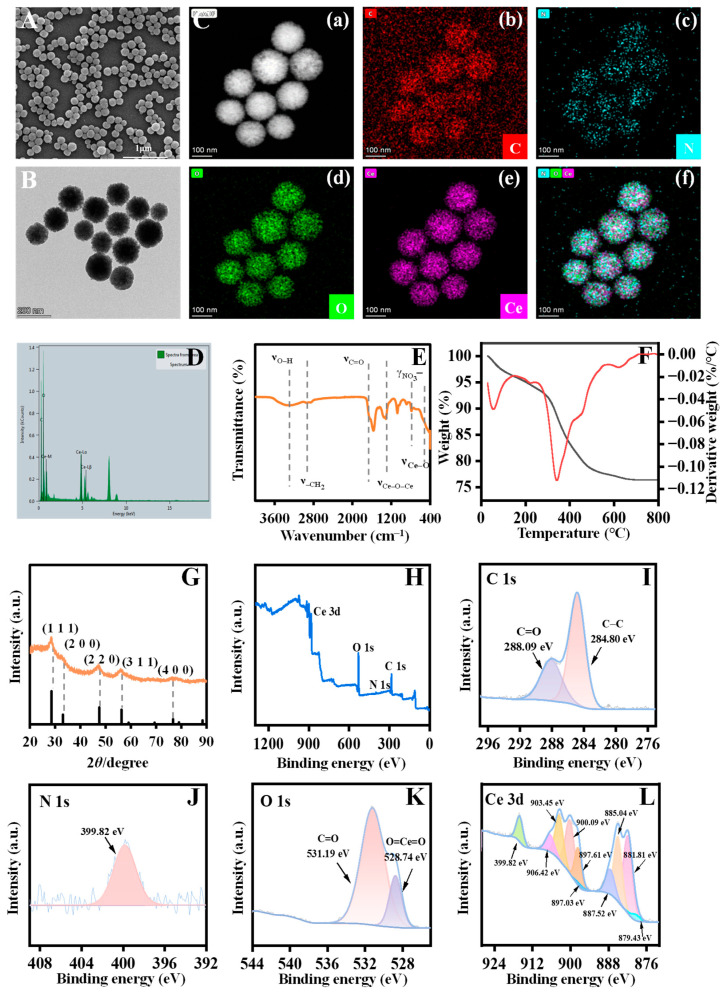
SEM images (**A**), TEM images (**B**), EDX results (**C**,**D**) (full EDS spectrum (**a**); elemental maps of C (**b**), N (**c**), O (**d**), and Ce (**e**); EDS map containing N, O, and Ce simultaneously (**f**)), FT-IR spectra (**E**), TGA curves (**F**), XRD patterns (**G**), and XPS results (**H**) for C 1s (**I**), N 1s (**J**), O 1s (**K**), and Ce 3d (**L**) of CeO_2_ NFs.

The morphological characteristics of the synthesized CeO_2_ NFs were characterized using FE-SEM. As exhibited in Figure 2A, the CeO_2_ NFs exhibit a regular spherical globular morphology with uniform particle size, averaging approximately 160 nm. The TEM results (Figure 2B) further indicate that the CeO_2_ NFs exhibit a distinct hydrangea flower-like structure, with the homogeneous distribution of C, N, O, and Ce elements (Figure 2C,D). The FT-IR spectrum (Figure 2E) indicates that the absorption peak at 3271 cm^−1^ is attributable to the stretching vibration of O–H bonds. The series of absorption peaks observed at 2958 cm^−1^, 1642 cm^−1^, and 786 cm^−1^ are attributed to the stretching vibrations of –CH_2_ and C=O, and the out-of-plane bending vibration of NO_3_^−^, respectively. This indicates that the CeO_2_ was surface-modified by PVP in situ during the synthesis process [36,37,38]. Two more bands are observed at 1311 cm^−1^ and 473 cm^−1^, corresponding to the stretching vibrations of Ce–O–Ce and Ce–O, respectively. This confirms the formation of CeO_2_ [39]. Figure 2G presents the XRD patterns of CeO_2_ NFs, with characteristic diffraction peaks at 2*θ* values of 28.6°, 32.5°, 47.1°, 56.1°, and 76.8°, corresponding to the (1 1 1), (2 0 0), (2 2 0), (3 1 1), and (4 0 0) crystal planes [40], respectively, which confirm that the CeO_2_ NFs adopt a cubic fluorite structure. The TGA results (Figure 2F) reveal that the primary weight loss occurs around 320 °C (~18%), primarily due to the decomposition of residual organic components, such as PVP [41]. The remaining material remains thermally stable at 500–800 °C, indicating excellent thermal stability of CeO_2_ NFs.

The surface elemental composition and oxidation states of the material were analyzed by XPS (Figure 2H–L). The survey spectrum (Figure 2H) exhibits four peaks at 285.24 eV, 400.23 eV, 530.93 eV, and 889.90 eV, corresponding to the elements C, N, O, and Ce, respectively, which is consistent with the TEM results. The C 1s spectrum (Figure 2I) displays characteristic peaks at 284.8 eV and 288.09 eV that are attributable to the C–C and C=O bonds [42], respectively. The N 1s spectrum (Figure 2J) shows a single peak at 399.82 eV, which corresponds to the amide/lactam nitrogen of the pyrrolidone ring in PVP. The O 1s spectrum (Figure 2K) features peaks at 531.19 eV and 528.74 eV that are assigned to C=O and C–O bonds or Ce–O, respectively [43]. The Ce 3d spectrum (Figure 2L) reveals six peaks at 881.81, 885.04, 897.61, 900.09, 903.45, and 916.01 eV corresponding to Ce(IV), and four peaks at 879.43, 887.52, 897.03, and 906.42 eV corresponding to Ce(III) [44]. And, based on the peak area, the relative contents were calculated to be about 80% and 20%, respectively, indicating that cerium predominantly exists as CeO_2_ within the material.

### 2.2. Optimization of Experimental Parameters

A systematic investigation was conducted to evaluate the effects of Ce(NO_3_)_3_·6H_2_O concentration, PVP amount, and synthesis time on the microstructure, crystalline phase, and Arg recognition performance of CeO_2_ NFs, aiming to determine the optimal preparation conditions. The influence of Ce(NO_3_)_3_·6H_2_O concentration was first examined, as the concentration of cerium ions is a critical factor in regulating nucleation kinetics and crystal growth rates. Under fixed reaction conditions (165 °C for 2 h and 0.45 g PVP), experiments were carried out for Ce(NO_3_)_3_·6H_2_O concentrations of 1.0, 1.2, 1.4, and 1.6 M. As shown in Figure S1A,B, at lower concentrations (1.0 M and 1.2 M) the CeO_2_ NFs exhibited non-uniform size distribution, which was attributed to the insufficient nucleation sites at low concentrations, leading to heterogeneous crystal growth. The corresponding XRD patterns (Figure S1F) reveal that the diffraction peaks of the (1 1 1), (2 0 0), (2 2 0), (3 1 1), and (4 0 0) crystal planes were weak and broadened, indicating poor crystallinity of the products. When the concentration was increased to 1.4 M, well-defined nanospheres with a regular morphology were obtained (Figure S1C). The XRD patterns show enhanced intensity and reduced full width at half maximum for all diffraction peaks, particularly for the (1 1 1) plane (Figure S1F), suggesting improved crystallinity. Concurrently, the FT-IR spectra (Figure S1E) exhibit markedly enhanced intensities for the stretching vibration of C=O (1646 cm^−1^) and out-of-plane bending vibration of NO_3_^−^ (786 cm^−1^). This indicates that sufficient precursor promotes uniform nucleation and crystal growth, with the PVP effectively encapsulating and guiding the crystal nuclei to self-assemble into spherical structures [40], thereby generating more CeO_2_ NFs. Upon further increasing the Ce(NO_3_)_3_·6H_2_O concentration to 1.6 M, the SEM images reveal a substantial increase in the nanosphere numbers (Figure S1D) of CeO_2_ NFs, yet severe agglomeration occurred. This may have been due to the explosive nucleation induced by the excessive Ce(NO_3_)_3_·6H_2_O concentration, preventing the PVP from adequately coating all the nascent nuclei and consequently triggering disordered aggregation. The FT-IR spectra (Figure S1E) show significantly enhanced intensities of characteristic peaks of C=O (1646 cm^−1^), Ce–O–Ce (1311 cm^−1^), and Ce–O (474 cm^−1^), confirming increased product yield. However, the XRD patterns display weakened and broadened diffraction peaks, indicating a deteriorated crystal quality and increased lattice defects. In addition, the fluorescence spectroscopy results demonstrate that the CeO_2_ NFs prepared at 1.4 M exhibited the highest *F*_L_/*F*_D_ ratio (where *F*_L_ and *F*_D_ represent the fluorescence intensities of the system after adding L-Arg and D-Arg, respectively) (Figure S1G,H), displaying optimal chiral recognition performance. Consequently, the Ce(NO_3_)_3_·6H_2_O concentration of 1.4 M was selected for subsequent experiments.

The effect of synthesis time was subsequently investigated. Under fixed conditions and the Ce(NO_3_)_3_·6H_2_O concentration at 1.4 M (0.45 g PVP, synthesis temperature was 165 °C), the influence of varying reaction times (1 h, 2 h, 3 h, and 4 h) on the CeO_2_ NFs was examined. The XRD analysis results reveal that, except for the material reacted for 1 h, which exhibited impurity peaks, all the other synthesized materials only displayed diffraction peaks corresponding to the (1 1 1), (2 0 0), (2 2 0), (3 1 1), and (4 0 0) crystal planes (Figure S2F), indicating a pure cubic fluorite structure. The TEM images visually show that the synthesis time can affect the morphology and size uniformity of the product. Insufficient crystal growth resulted in an inconsistent size distribution of the nanoparticles at 1 h reaction time (Figure S2A), but adequate nucleation and stable growth were achieved at 2–3 h reaction time (Figure S2B,C), yielding sufficient quantities of nanoparticles with uniform particle sizes. When the reaction time was extended to 4 h (Figure S2D), the Ostwald ripening effect became apparent [45], causing partial dissolution of smaller particles and enlargement of larger ones, resulting in decreased product yield. This aligns with the significant weakening of the characteristic peak intensities observed in the FT-IR spectra for C=O (1646 cm^−1^), Ce–O–Ce (1311 cm^−1^) and Ce–O (474 cm^−1^) (Figure S2E). In addition, the fluorescence measurements reveal an increasing trend in the *F*_L_/*F*_D_ ratio with an extended reaction time, peaking at 3 h (Figure S2G,H), which was selected as the optimized reaction time.

Finally, the influence of PVP amount on the synthesis of CeO_2_ NFs was evaluated by incrementally increasing the PVP dosage with the optimized Ce(NO_3_)_3_·6H_2_O concentration (1.4 M) and synthesis time (3 h) (synthesis temperature remained at 165 °C). The TEM results show that as the dosage of PVP increased from 0.3 g to 0.75 g, the particle size of CeO_2_ NFs gradually increased (Figure S3A–D), confirming the regulatory effect of PVP on the particle size. The underlying mechanism could be that the carbonyl groups in PVP molecules have an appropriate coordination ability with Ce^3+^/Ce^4+^, and can guide the anisotropic growth of crystals through selective adsorption on the specific crystal planes. The XRD patterns indicate that the adsorption effect of PVP mainly affects the growth rates of (1 1 1) and (2 0 0) crystal planes, resulting in significant changes in the intensity of their diffraction peaks (Figure S3F). The FT-IR spectra reveal that the characteristic peaks’ intensities, such as Ce–O–Ce (1311 cm^−1^) and Ce–O (474 cm^−1^), exhibited decreasing trends with an increasing PVP dosage (Figure S3E). This indicates that insufficient PVP fails to effectively coat the crystal nuclei, while excessive amounts overly suppress their growth and fusion. In addition, the fluorescence performance testing shows that when the PVP dosage was 0.55 g, the material had the strongest recognition ability for Arg enantiomers (Figure S3G,H).

In summary, the concentration of cerium ions primarily regulates the nucleation process and product yield; the synthesis time determines the crystals’ crystallinity and integrity; while PVP, acting as a structural directing agent, significantly influences the particle size and dispersion of the product. Through this systematic investigation, CeO_2_ NFs exhibiting uniform morphology, moderate crystallinity, and excellent chiral recognition performance towards Arg were successfully prepared under optimized conditions of 1.4 M of Ce(NO_3_)_3_·6H_2_O, 0.55 g of PVP, and a 3 h synthesis time.

Furthermore, a systematic optimization of experimental conditions was performed to obtain the maximum fluorescence selectivity to the Arg enantiomer, including the dilution factor of CeO_2_ NFs, buffer pH, the concentrations of H_2_O_2_ and OPD, incubation temperature, and reaction time. Figure S4A shows the fluorescence intensity ratio (*F*_L_/*F*_D_) at 560 nm for the L-Arg and D-Arg reaction systems (CeO_2_ NFs + H_2_O_2_ + OPD + L/D-Arg) under different dilution multiples of CeO_2_ NFs. It is obvious that the maximum *F*_L_/*F*_D_ was achieved at a dilution multiple of 24, which was selected for subsequent experiments. The buffer pH emerged as another critical factor influencing the POD-like activity of CeO_2_ NFs. Within the tested range of pH 3 to 11, the highest *F*_L_/*F*_D_ value at 560 nm was recorded at pH 10.0, indicating optimal recognition ability of CeO_2_ NFs for Arg enantiomers (Figure S4B). When the H_2_O_2_ concentration was increased from 4.76 mM to 28.53 mM, the difference in the *F*_L_/*F*_D_ values at 560 nm was not significant. It reached the maximum ratio at 14.27 mM (initial concentration of 10 M), which was selected as the optimized condition for subsequent studies (Figure S4C). Additionally, the fluorescence intensity ratio varied with the OPD concentration from 1.07 mM to 6.42 mM, reaching the maximum value at 2.14 mM (initial concentration of 5 mM), which was used for further experiments (Figure S4D). As the reaction temperature increased from 35 °C to 65 °C, the fluorescence ratio at 560 nm varied, with *F*_L_/*F*_D_ reaching its maximum at 45 °C, which was chosen for subsequent experiments (Figure S4E). Moreover, the *F*_L_/*F*_D_ value of the reaction system increased with an increase in the reaction time from 1 min to 4 min, peaking at 4 min, which was used for further analysis (Figure S4F). In summary, the optimized conditions for the recognition of Arg enantiomers are: a dilution multiple of 24; buffer pH at 10; H_2_O_2_ and OPD concentrations of 14.27 mM and 2.14 mM, respectively; incubation temperature of 45 °C; and reaction time of 4 min.

In addition, to obtain the best response for *p*-NP and ALP detection through fluorescence analysis, the effects of buffer pH, the dilution multiple of CeO_2_ NFs, incubation temperature, 4-MUP concentration, and reaction time were investigated. The maximum fluorescence intensity at 450 nm was observed with a buffer pH of 9 (Figure S5A) (pH range of 2–11). This pH dependence reflects the Lewis acidity of Ce^4+^ sites: acidic conditions protonate surface oxygens, weakening substrate binding, while strongly alkaline conditions (>10) may cause deprotonation of 4-MU or competition with OH^−^. Figure S5B exhibits the variance in the fluorescence intensity of the reaction system with the CeO_2_ NFs dilution multiple ranging from 5 to 40, and the optimal signal was achieved at a dilution multiple of 10, which was subsequently employed for further experiments. Lower dilution (higher nanozyme concentration) may cause aggregation or inner filter effects, whereas higher dilution reduces active site availability. Additionally, the fluorescence intensity increased markedly as the incubation temperature increased from 35 °C to 65 °C (Figure S5C), indicating an endothermic catalytic process. Considering the instrument’s detection limits, 55 °C was selected for subsequent experiments. The influence of 4-MUP concentration was examined within the range of 46.9–187.5 μM (initial concentration from 156.2 μM to 625.0 μM). The fluorescence intensity increased with the 4-MUP concentration up to 93.7 μM (initial concentration of 312.5 μM) and then plateaued (Figure S5D), following typical Michaelis–Menten saturation kinetics. The plateau indicates that the active sites of CeO_2_ NFs became saturated at higher substrate concentrations. The concentration of 93.7 μM (still below the inhibitory range) was chosen to ensure a strong enough signal but avoid substrate-induced quenching. Finally, the reaction time was optimized (Figure S5E). The fluorescence intensity at 450 nm increased from 1 min to 3.5 min and plateaued thereafter, so 3.5 min was used as the optimized reaction time. In conclusion, the optimum conditions for *p*-NP and ALP detection are buffer pH of 9, CeO_2_ NFs dilution multiple of 10, incubation temperature of 55 °C, 4-MUP concentration of 93.7 μM, and reaction time of 3.5 min.

### 2.3. Enzymatic Activity of CeO_2_ NFs

**Figure 3:**
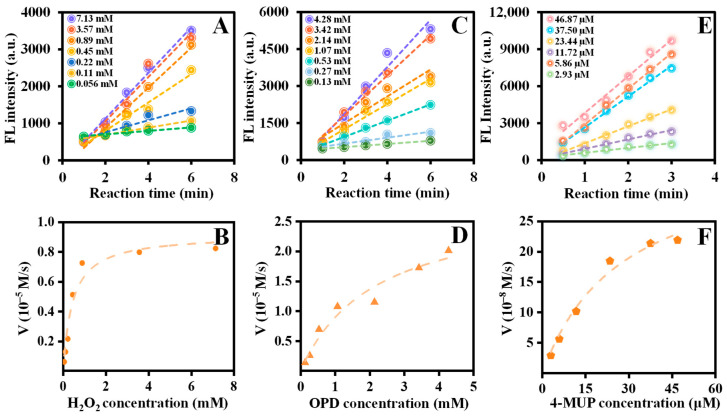
The linear relationship between fluorescence intensity and reaction time (within 6 min) with H_2_O_2_ concentrations of 0.056, 0.11, 0.22, 0.45, 0.89, 3.57, and 7.13 mM (**A**) and Michaelis–Menten plot (**B**) based on the POD-like activity of CeO_2_ NFs. The linear relationship between fluorescence intensity and reaction time (within 6 min) with OPD concentrations of 0.13, 0.27, 0.53, 1.07, 2.14, 3.42, and 4.28 mM (**C**) and Michaelis–Menten plot (**D**) based on the POD-like activity of CeO_2_ NFs. The linear relationship between fluorescence intensity and reaction time (within 6 min) with 4-MUP concentrations of 2.93, 5.86, 11.72, 23.44, 37.50, and 46.87 μM (**E**) and Michaelis–Menten plot (**F**) based on the hydrolase-like activity of CeO_2_ NFs.

Through an enzymatic kinetic analysis, a systematic quantitative evaluation of the catalytic performance of CeO_2_ NFs was conducted. For its POD-like activity, the steady-state kinetic parameters were determined using the initial reaction rate method, with OPD and H_2_O_2_ as substrates. Based on the Michaelis–Menten equation (*V* = *V*_max_[*S*]/(*K*_m_ + [*S*]), Origin 2021 software was used to perform nonlinear fitting of the reaction rates at different H_2_O_2_ and OPD concentrations (as shown in Figure 3B,D), yielding corresponding *K*_m_ values of 0.44 mM (H_2_O_2_) and 2.17 mM (OPD), and *V*_max_ values of 917.72 × 10^−8^ M/s (H_2_O_2_) and 2849.45 × 10^−8^ M/s (OPD). Table 1 shows the performance comparison of CeO_2_ NFs with natural horseradish peroxidase (HRP) and other POD mimics. Its significantly higher *V*_max_ value proves that the CeO_2_ NFs have an excellent catalytic reaction rate.

Similarly, the hydrolase-like activity of CeO_2_ NFs was evaluated using 4-MUP as the substrate. As shown in Figure 3F, Origin 2021 software was used to perform nonlinear fitting of the reaction rates at different concentrations of 4-MUP, obtaining *K*_m_ and *V*_max_ values of 27.93 μM and 36.63 × 10^−8^ M/s, respectively. As shown in Table 2, the *V*_max_ value of CeO_2_ NFs is significantly higher than that of other similar hydrolase mimetics, indicating an excellent catalytic reaction rate. However, its *K*_m_ value is relatively high, implying that its substrate affinity is weaker than that of natural ALP and some previously reported mimetic enzymes. Nevertheless, the high *V*_max_ still indicates a certain application potential of CeO_2_ NFs to enzyme-catalyzed sensing and detection.

### 2.4. Chiral Recognition of Arg Enantiomers and Mechanism Study

**Figure 4:**
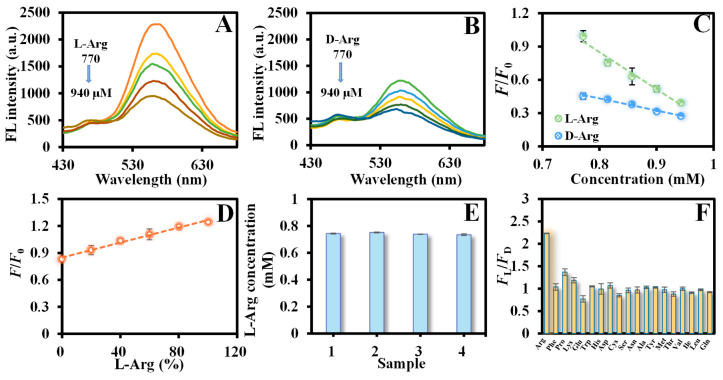
Fluorescence emission spectra of the system (CeO_2_ NFs + H_2_O_2_ + OPD) with varied L-Arg concentrations (770, 810, 860, 900, and 940 μM) (**A**) and varied D-Arg concentrations (770, 810, 860, 900, and 940 μM) (**B**). Linear relationship between fluorescence intensities and L/D-Arg concentrations (**C**). Exploration of enantiomeric excess (**D**). L-Arg detection results in food-grade arginine samples (**E**). The fluorescence response ratios of CeO_2_ NFs to different AA enantiomers (**F**). All error bars represent the standard deviation (SD) from three independent replicates (*n* = 3).

The addition of L/D-Arg at varying concentrations (770–940 μM) resulted in a concentration-dependent quenching of the fluorescence of the reaction system (CeO_2_ NFs + H_2_O_2_ + OPD), changing it from an “on” to “off” state. As depicted in Figure 4A,B, a stark contrast was observed between the enantiomers; L-Arg caused a marked reduction in the fluorescence intensity (Figure 4A), whereas D-Arg produced only an unobvious change under identical conditions (Figure 4B).

As shown in Figure 4C, within the L/D-Arg concentrations of 770–940 μM, the fluorescence ratio *F*/*F*_0_ (where *F*_0_ and *F* represent the fluorescence intensities without added AAs and with added L/D-Arg, respectively) exhibits good linear correlations with the L/D-Arg concentrations, with regression equations of y = −3.3374 x + 3.5194 (R^2^ = 0.9720) (L-Arg) and y = −1.0881x + 1.3025 (R^2^ = 0.9843) (D-Arg), and the limits of detection (LOD = 3σ/S, where σ represents the standard deviation of blank measurements and S denotes the slope of the calibration curve) (*n* = 11) calculated to be 26.00 μM (L-Arg) and 79.00 μM (D-Arg). Compared to previously reported methods (Table 3), this method offers comparable sensitivity and better enantiomeric selectivity, with ef value as high as 2.48. The enantiomeric excess recognition ability of CeO_2_ NFs was systematically investigated through adjusting the percentage of L-Arg in the mixture. The corresponding calibration curve (Figure 4D) demonstrates an excellent linear response (y = 0.0042x + 0.8487, R^2^ = 0.9892) of fluorescence ratio (*F*/*F*_0_) to the L-Arg fraction. Therefore, the developed platform can not only discriminate Arg enantiomers but also precisely quantify the enantiomeric excess.

To evaluate the feasibility of the developed method for practical applications, quantitative analyses of real samples were conducted under optimized conditions using the standard addition method. As shown in Table S1, the recovery rates in rabbit plasma for the target analytes ranged from 94.7% to 108.1%, with relative standard deviations (RSDs) below 5.0%. Furthermore, the L-Arg content was determined in commercially available food-grade amino acid samples (Figure 4E). The concentration values from four parallel experiments exhibited high consistency, with a relative error of 2.0% compared to the minimum concentration specified. In addition, four independent batches of CeO_2_ NFs were synthesized, and their fluorescence responses related to POD-like activity were measured in triplicate. The results (Figure S6A) indicate that the material exhibits good batch-to-batch reproducibility. The above results indicate that the method exhibits high accuracy and repeatability, presenting great potential for practical application to real sample analysis.

To evaluate the selective recognition ability of CeO_2_ NFs towards AA enantiomers, the fluorescence response ratio *F*_L_/*F*_D_ of the system in the presence of different amino acid enantiomers was measured, including Arg, phenylalanine (Phe), proline (Pro), lysine (Lys), glutamic acid (Glu), Trp, histidine (His), aspartic acid (Asp), cysteine (Cys), serine (Ser), asparagine (Asn), Ala, tyrosine (Tyr), methionine (Met), threonine (Thr), Valine (Val), isoleucine (Ile), leucine (Leu), and glutamine (Gln). Notably, as shown in Figure 4F, the other AAs and their enantiomers all exhibited extremely low *F*_L_/*F*_D_ values; only Arg showed a significantly elevated *F*_L_/*F*_D_ ratio, which indicates that the CeO_2_ NFs have a significant selective recognition ability towards L/D-Arg.

**Figure 5:**
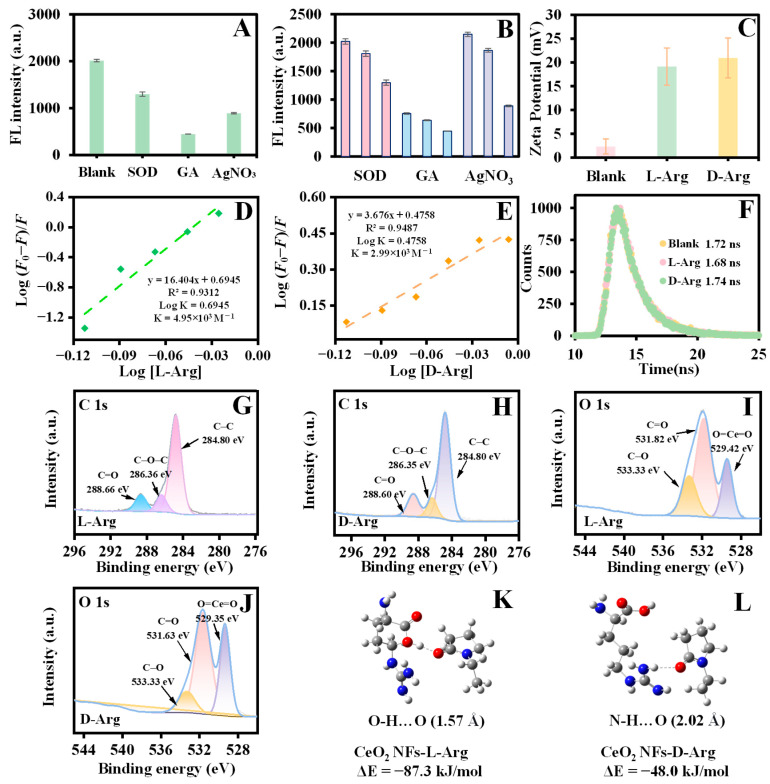
Effects of various active scavengers (**A**) with different concentrations (**B**) on catalytic process. Zeta potential of CeO_2_ NFs/H_2_O_2_/OPD with L/D-Arg (**C**). Linear fitting curves between fluorescence intensity ratio (Log(*F* − *F*_min_)/(*F*_max_ − *F*)) and Log[L-Arg] (**D**) and Log[D-Arg] (**E**). Fluorescence lifetime spectrum of CeO_2_ NFs/H_2_O_2_/OPD with L/D-Arg (**F**). XPS spectrum of C 1s of CeO_2_ NFs + L-Arg (**G**) and CeO_2_ NFs + D-Arg (**H**), and O 1s of CeO_2_ NFs + L-Arg (**I**) and CeO_2_ NFs + D-Arg (**J**). DFT calculation results for CeO_2_ NFs with L-Arg (**K**) and D-Arg (**L**). Blank: CeO_2_ NFs + H_2_O_2_ + OPD. All error bars represent standard deviation (SD) from three independent replicates (*n* = 3).

The catalytic mechanism of CeO_2_ NFs as POD mimics and the principle of chiral recognition for Arg enantiomers were investigated. Free radical trapping experiments confirmed the presence of reactive species involved in the catalytic process. As shown in Figure 5A,B, the fluorescence intensity of reaction solution (CeO_2_ NFs + H_2_O_2_ + OPD) significantly decreased with the addition of superoxide dismutase (SOD) (scavenge superoxide anion radicals (O_2_^•−^)), gallic acid (GA) (scavenge singlet oxygen (·^1^O_2_)) and AgNO_3_ (clear the electronics). Furthermore, with increasing concentrations of SOD, GA, and AgNO_3_, the fluorescence intensity of the reaction system at 560 nm markedly decreased, indicating that O_2_^•−^ and ∙^1^O_2_ may be the key reactive intermediates in the catalytic process, playing a crucial role in the reaction’s progress. The POD-like activity of CeO_2_ NFs may originate from the Ce^3+^/Ce^4+^ redox cycle on its surface, which facilitates electron transfer between H_2_O_2_ and another substrate, OPD. The zeta potential measurements (Figure 5C) reveal values of +2.35 mV, +19.11 mV, and +20.94 mV for the CeO_2_ NFs/H_2_O_2_/OPD, CeO_2_ NFs/H_2_O_2_/OPD + L-Arg, and CeO_2_ NFs/H_2_O_2_/OPD + D-Arg, respectively. The significant increase in potential indicates that the positively charged L/D-Arg molecules were successfully adsorbed onto the CeO_2_ NFs’ surfaces via electrostatic interactions. This adsorption likely facilitated electron transfer between the CeO_2_ NFs and Arg. Under excited-state conditions, this interaction intensified, ultimately leading to fluorescence quenching of CeO_2_ NFs through efficient intermolecular collisions.

The fluorescence lifetime measurements (Figure 5F) show no significant change (Δτ < 5%), indicating that the quenching process belongs to a static quenching mechanism. Based on the formula $\log[(F_{0}-F)/F]=\log K+n\log\left[L/\mathrm{D-Arg}\right]$, the apparent binding constant of CeO_2_ NFs with L-Arg at 45 °C is 4.95 × 10^3^ M^−1^ (Figure 5D), and with D-Arg it is 2.99 × 10^3^ M^−1^ (Figure 5E). The comparison of binding constants suggests a stronger molecular affinity between the CeO_2_ NFs and L-Arg [63]. The XPS analysis shows that after adding L/D-Arg, new characteristic peaks appear at 286.36 eV and 533.33 eV in the C 1s (Figure 5G,H) and O 1s (Figure 5I,J) spectra, belonging to the C–O bond, confirming the formation of new carbon–oxygen single bonds in the system. When comparing the O 1s spectra after adding L-Arg and D-Arg, it is notable that the relative content of O=Ce=O in the CeO_2_ NFs + D-Arg system is significantly higher than that in the CeO_2_ NFs + L-Arg system, which indicates that more L-Arg is adsorbed on the surface of the material and interacts with its –COOH group. To further study the interaction between the CeO_2_ NFs and L-Arg/D-Arg, density functional theory (DFT) calculations were performed to analyze the ground-state structures of different systems (see Supporting Information for more details) [64,65]. The calculation results (Figure 5K,L) show that the binding energy of CeO_2_ NFs with L-Arg is −87.3 kJ/mol, and with D-Arg it is −48.0 kJ/mol. The binding energy between the CeO_2_ NFs and L/D-Arg is negative, serving as a direct indicator of their thermodynamically stable complex formation. Meanwhile, the significant energy difference (39.3 kJ/mol) and the shorter hydrogen bond length between the two systems (CeO_2_ NFs-L-Arg: O-H…O (1.57 Å); CeO_2_ NFs-D-Arg: N-H…O (2.02 Å)) indicate that the stereoisomer configuration of L-Arg shows a better affinity with the CeO_2_ NFs and is more likely to undergo binding reactions. Consequently, the interaction between the CeO_2_ NFs and L-Arg is stronger than that with D-Arg. These results explain the experimental observation that CeO_2_ NFs can discriminate Arg enantiomers.

### 2.5. Detection of p-NP and ALP Based on “On-Off-On” Fluorescence Sensor

**Figure 6:**
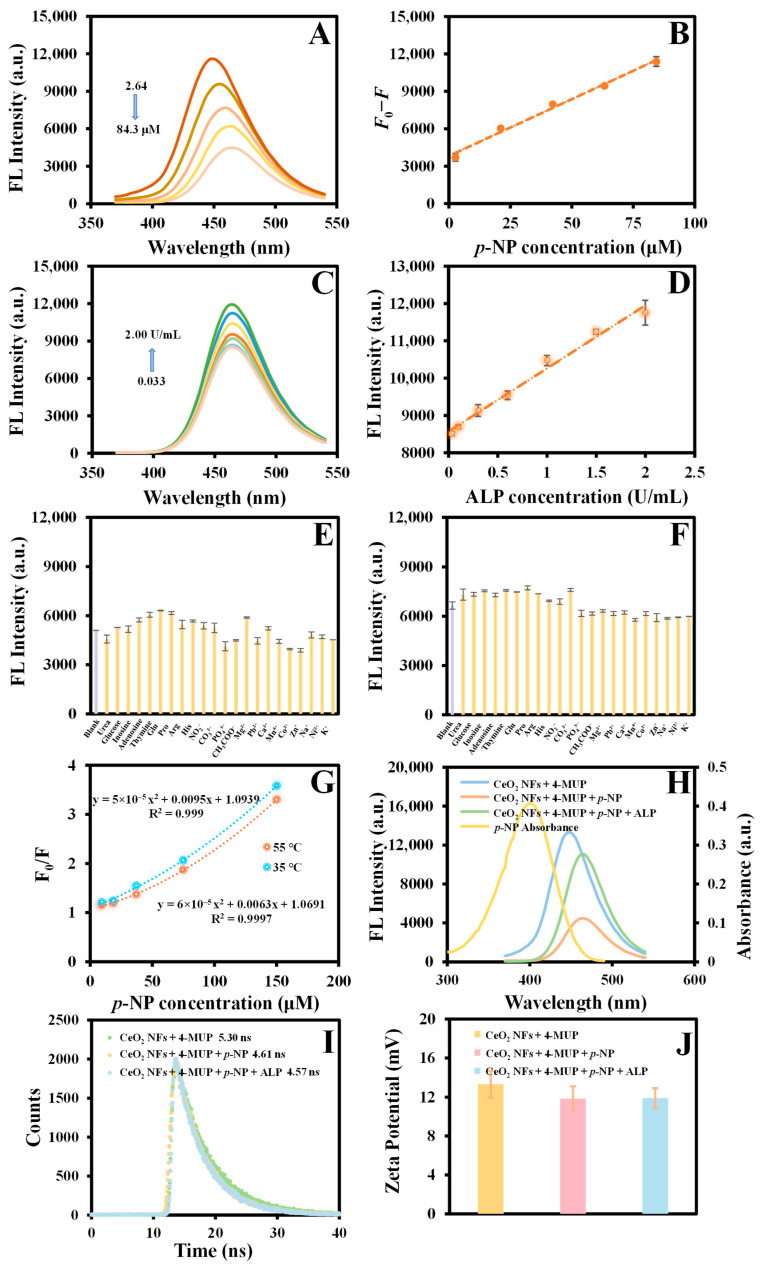
Fluorescence emission spectra of the system (CeO_2_ NFs + 4-MUP) with varied *p*-NP concentrations (2.64, 21.1, 42.2, 63.3, and 84.3 μM) (**A**). Linear relationship between fluorescence intensities and *p*-NP concentrations (**B**). Fluorescence emission spectra of the system (CeO_2_ NFs + 4-MUP + *p*-NP) with varied ALP concentrations (0.033, 0.10, 0.30, 0.60, 1.00, 1.50, and 2.00 U/mL) (**C**). Linear relationship between fluorescence intensities and ALP concentrations (**D**). The influence of different interferent substances on the reaction solution with the addition of *p*-NP ((**E**), Blank1: 5105) and *p*-NP + ALP ((**F**), Blank2: 6647). Stern–Volmer plot (**G**). Emission spectra and UV-Vis absorption spectrum of different solutions (**H**). Fluorescence lifetime spectra of CeO_2_ NFs + 4-MUP, CeO_2_ NFs + 4-MUP + *p*-NP, and CeO_2_ NFs + 4-MUP + *p*-NP + ALP (**I**). Zeta potential of CeO_2_ NFs + 4-MUP, CeO_2_ NFs + 4-MUP + *p*-NP, and CeO_2_ NFs + 4-MUP + *p*-NP + ALP (**J**). All error bars represent the standard deviation (SD) from three independent replicates (*n* = 3).

The hydrolase-like activity of CeO_2_ NFs can catalyze 4-MUP to generate 4-MU, exhibiting an obvious fluorescence emission at 448 nm. The introduction of *p*-NP causes a significant quenching of the fluorescence intensity, effectively turning the signal from the “on” to “off” state. The further addition of ALP to the reaction system (CeO_2_ NFs + 4-MUP + *p*-NP) leads to a significant recovery of fluorescence intensity. By adjusting the ALP concentration, a gradient recovery of the fluorescence signal can be achieved, thereby enabling a switch from the fluorescence “off” to “on” state, which can be used for *p*-NP and ALP detection. As exhibited in Figure 6A,B, the fluorescence intensity of the system (CeO_2_ NFs + 4-MUP) decreases with an increase in the *p*-NP concentrations. For concentrations ranging from 10.0 to 84.3 μM, the fluorescence intensity difference (*F*_0_ − *F*; *F*_0_ and *F* represent the fluorescence intensities of the system in the absence and presence of *p*-NP, respectively) shows a good linear relationship with the *p*-NP concentration, and the linear regression equation is y = 91.12x + 3827.4 (R^2^ = 0.9904), with an LOD of 7.07 μM. Under the fixed initial concentration of *p*-NP (1 mM), the fluorescence intensity of the system (CeO_2_ NFs + 4-MUP + *p*-NP) gradually increases with rising ALP concentrations, varying from 0.30 to 2.00 U/mL (Figure 6C). A good linear equation is obtained, y = 1691.2x + 8572.5 (R^2^ = 0.9874) (Figure 6D), with an LOD of 200 mU/mL. This method exhibits considerable sensitivity compared to other reported methods for detecting *p*-NP and ALP (Table 4 and Table 5), and it stands out as one of the few techniques that can achieve the dual purpose of detecting *p*-NP and ALP in a single system by controlling the switching of fluorescence signal.

To further validate the reliability of the developed method for *p*-NP and ALP detection in real samples, spiked recovery assays were conducted using rabbit plasma (ALP) and water samples (*p*-NP) from Yun Lake, Jin Lake, and tap water. As exhibited in Tables S2 and S3, the recovery rates for the target analytes ranged from 80.6% to 103.6%, with RSD values below 5.0%. These results indicate that the method exhibits high accuracy and reliability for *p*-NP and ALP detection, presenting great potential for practical application in sample analysis. By investigating the effects of various potential interfering substances on the detection of *p*-NP and ALP, the interference-resistant ability of the CeO_2_ NFs + 4-MUP sensing system was evaluated. As shown in Figure 6E,F, the influence of the vast majority of tested substances (such as common alkaloids, AAs, metal ions, etc.) on the fluorescence intensity was negligible compared to the blank ones. The relative standard deviation (RSD) of hydrolase-like activity between the different batches was less than 5%, indicating that the CeO_2_ NFs exhibit good reproducibility (Figure S6B). These results confirm that the sensing platform has excellent interference performance and outstanding matrix tolerance, laying the foundation for its accurate detection of *p*-NP and ALP in real samples.

In this sensing system, the CeO_2_ NFs catalyze the hydrolysis of 4-MUP to produce highly fluorescent 4-MU, which serves as the signal reporter. *p*-NP is then quantified based on its role as an efficient quencher of 4-MU fluorescence. When analyzing the Stern–Volmer relationship for the CeO_2_ NFs + 4-MUP + *p*-NP system, a second-order polynomial curve provides the best fit (Figure 6G), strongly indicating the coexistence of both static and dynamic quenching mechanisms [66]. To elucidate the quenching mechanism of *p*-NP on 4-MU fluorescence, the UV-Vis absorption spectra and corresponding fluorescence lifetimes were measured. The emission spectra associated with the CeO_2_ NFs and the UV-Vis absorption spectrum of *p*-NP are presented in Figure 6H. The results indicate substantial overlap between the UV-Vis absorption spectrum of *p*-NP and the emission spectra of CeO_2_ NFs + 4-MUP, accompanied by a 15 nm bathochromic shift in the 4-MU emission upon *p*-NP addition. Therefore, the internal filtering effect (IEF) is identified as the potential quenching mechanism. Fluorescence lifetime measurements were further employed to distinguish the type of quenching (Figure 6I). The fluorescence lifetime of the CeO_2_ NFs + 4-MUP + *p*-NP system (4.61 ns) exhibited a notable decrease compared to the CeO_2_ NFs + 4-MUP system (5.30 ns) (Δτ > 5%), providing strong support for dynamic quenching. The zeta potential analysis (Figure 6J) indicated that the surface potential slightly changed after the addition of *p*-NP and ALP, which indicates that electrostatic interactions play a tiny role in the catalytic process. Thus, the fluorescence attenuation results from the combined effect of dynamic and static quenching mechanisms. The dynamic component is mediated by collisional energy transfer, while the static component is facilitated by the electrostatically assisted formation of ground-state complexes.

**Table 4:** Comparisons with the previously reported methods for the detection of *p*-NP.

Probe	Detection Range (μM)	LOD/LOQ (μM)	Ref.
GSH-CuNCs	0.1–150	0.02/0.10	[67]
Ag NCs	5–140	1.28/5.00	[68]
β-CD-CdTe	20–100	0.30/20	[69]
CDs	0.2–20	0.069/0.20	[70]
β-CD@ZnO QDs	1–40	0.34/1.00	[71]
CTAB-Cu NPs	0.83–125 (FL) 6.67–600 (UV-Vis)	0.18/0.83 4.97/6.67	[72]
CeO_2_ NFs	10.0–84.3	7.07/10.0	This work

Abbreviations: GSH-CuNCs, glutathione-stabilized copper nanoclusters; Ag NCs, Ag nanoclusters; β-CD-CdTe, β-Cyclodextrin-capped CdTe quantum dots; CDs, carbon dots; β-CD@ZnO QDs, β-Cyclodextrin-capped zinc oxide quantum dots; CTAB-Cu NPs, cetyltrimethylammonium bromide-stabilized copper nanoparticles.

**Table 5:** Comparisons with the previously reported methods for the detection of ALP.

Probe	Detection Range (mU/mL)	LOD/LOQ (mU/mL)	Ref.
GSH-CuNCs	0.01–40	0.003/0.01	[67]
Pt/HOFs	0.5–8	0.46/0.50	[73]
Ln-CPs	0.1–6	0.026/0.10	[74]
Cu-Cy	1–100	0.10/1.00	[75]
Fe/C NS	0.05–6	0.03/0.05	[76]
SiQD	0.02–2.0	0.015	[77]
CeO_2_ NFs	300–2000	200/300	This work

Abbreviations: GSH-CuNCs, glutathione-stabilized copper nanoclusters; Pt/HOFs, Pt nanoparticle-functionalized hydrogen-bonded organic frameworks; Ln-CPs, gelatinous Ln^3+^-coordination polymers; Cu-Cy, two-dimensional sheet-like copper–cysteamine fluorescent probe; Fe/C NS, Fe-doped carbon nanosheet; SiQD, silicon quantum dots.

## 3. Materials and Methods

### 3.1. Materials and Reagents

The reagents, chemicals, and instruments are described in the Supplementary Materials.

### 3.2. Preparation of CeO_2_ NFs

A total of 15 mL of ethylene glycol (EG) was added to a flask and heated at 165 °C in an oil bath for 15 min (A). A total of 0.55 g of PVP was dissolved in 36 mL of EG, then 50 μL of 1 M NaCl solution and 1.8 mL of 1.4 M Ce (NO_3_)_3_·6H_2_O solution were sequentially added with stirring (B). Under continuous magnetic stirring, solution B was slowly dropwise added into solution A, then the mixture was heated in an oil bath at 165 °C to maintain the reaction for 3 h, followed by washing three times with methanol and centrifuged using a high-speed centrifuge for 10 min at 10,000 rpm (6745× *g*). Finally, the obtained product was dispersed in 2 mL of methanol before use.

### 3.3. Chiral Recognition and Detection of Arg Enantiomer

A total of 100 μL of CeO_2_ NFs (diluted 24 fold), 300 μL of L/D-Arg solutions with different concentrations (770–940 μM), 300 μL of OPD (5 mM, pH 10.0), and 1 μL of H_2_O_2_ (10 M) were added into a 2.0 mL centrifuge tube, which then was incubated at 45 °C for 4 min. The fluorescence intensity was measured at 560 nm with excitation of 365 nm. Each sample was measured three times.

### 3.4. Detection of p-NP and ALP

For *p*-NP detection, 100 μL of CeO_2_ NFs (tenfold dilution), 300 μL of 4-MUP (0.3125 mM), 300 μL of *p*-NP with different concentrations (87–280 μM), and 300 μL of HEPES buffer solution (10 mM, pH 9.0) were individually added into a 2.0 mL centrifuge tube, and then incubated at 55 °C for 3.5 min. The fluorescence intensity was measured at an excitation wavelength of 350 nm and emission wavelength of 460 nm. For ALP activity detection, 100 μL of CeO_2_ NFs (tenfold dilution), 300 μL of 4-MUP (0.3125 mM), 300 μL of *p*-NP (0.28 mM), 300 μL of HEPES buffer (10 mM, pH 9.0), and 1 μL of ALP with different concentrations (300–2000 mU/mL) were added into a 2.0 mL centrifuge tube and incubated at 55 °C for 3.5 min. The fluorescence intensity was measured at an excitation wavelength of 350 nm and emission wavelength of 460 nm. Each sample was measured three times.

### 3.5. Enzymatic Kinetics Study

The POD-like activity of CeO_2_ NFs was analyzed by catalyzing OPD to generate DAP in the presence of H_2_O_2_, with varying concentrations of OPD (0.3–10 mM) and H_2_O_2_ (0.039–5 M). Initially, the concentration of H_2_O_2_ was fixed at 10 M. A total of 100 μL of CeO_2_ NFs (diluted 24 fold), 300 μL of buffer solution (pH 10.0), 300 μL of OPD with different concentrations (0.3–10 mM), and 1 μL of H_2_O_2_ were added into a 2.0 mL centrifuge tube, which then was incubated at 45 °C for 1, 2, 3, 4, 6, 8, 10, 15, and 20 min, respectively. Correspondingly, the concentration of OPD was fixed at 5 mM. A total of 100 μL of CeO_2_ NFs (diluted 24 fold), 300 μL of buffer solution (pH 10.0), 300 μL of OPD, and 1 μL of H_2_O_2_ with different concentrations (0.039–5 M) were added into a 2.0 mL centrifuge tube, which then was incubated at 45 °C for 1, 2, 3, 4, 6, 8, 10, 15, and 20 min, respectively. The fluorescence intensity was measured at 560 nm with excitation wavelength of 365 nm. Each sample was measured three times.

The hydrolase-like activity of CeO_2_ NFs was evaluated at varying substrate (4-MUP) concentrations ranging from 9.8 to 156.2 μM, with the reaction time varying from 0.5 min to 3.5 min. A total of 100 μL of CeO_2_ NFs (tenfold dilution), 300 μL of 4-MUP with different concentrations (9.8–156.2 μM), and 600 μL of HEPES buffer solution (10 mM, pH 9.0) were individually added into a 2.0 mL centrifuge tube, which was then incubated at 55 °C for 0.5, 1, 1.5, 2, 2.5, 3, and 3.5 min, respectively. The fluorescence intensity was measured at 460 nm with excitation wavelength of 350 nm. Each sample was measured three times. Based on the Michaelis–Menten kinetic model—*V* = *V*_max_[*S*]/(*K*_m_ + [*S*]) (where *V* represents the initial reaction rate, *V*_max_ represents the maximum reaction rate, [*S*] is the substrate concentration, and *K*_m_ is the Michaelis–Menten constant)—Origin 2021 software was used to perform nonlinear fitting of the reaction rates at different substrate concentrations, thereby obtaining the corresponding *K*_m_ and *V*_max_.

## 4. Conclusions

In summary, this study successfully constructed a multifunctional fluorescence sensing platform based on CeO_2_ NFs, which were surface-modified by PVP. By ingeniously leveraging the synergistic effect of the unique dual catalytic activities (POD- and hydrolase-like activities) of CeO_2_ NFs, the platform not only achieved the high-selectivity chiral recognition of arginine enantiomers (enantiomeric factor ef = 2.48), but also established an “on-off-on” logic gate sensing mode for the detection of *p*-NP and ALP. The experimental results demonstrate that the proposed sensor possesses excellent selectivity, stability, and anti-interference capability, and can successfully achieve sensitive detection of L-Arg, *p*-NP, and ALP in complex biological samples. Nevertheless, the platform’s long-term storage stability and practical applicability to complex matrices require further evaluation. Future work will focus on addressing these issues and exploring the immobilization of nanozymes to construct reusable sensors.

## Figures and Tables

**Table 1:** The *K*_m_ and *V*_max_ of different materials using H_2_O_2_ and OPD as the substrate.

Catalyst	H_2_O_2_		OPD		Ref.
	*K*_m_ (mM)	*V*_max_ (10^−8^ M/s)	*K*_m_ (mM)	*V*_max_ (10^−8^ M/s)	
HRP	0.34	9.48	0.59	4.65	[46]
AgPt	76.05	12,849.00	0.13	8971.00	[47]
Au_1_Ag_4_Pd_1_	0.52	6.37	10.61	19.20	[48]
AgNPs@Ti_3_C_2_ NSs	22.20	18.20	0.26	43.20	[49]
IrO_2_/rGO nanocomposites	229.00	372.90	0.61	31.30	[50]
CeO_2_ NFs	0.44	917.72	2.17	2849.45	This work

Abbreviations: HRP, horseradish peroxidase; AgPt, AgPt bimetallic nanoparticles; Au_1_Ag_4_Pd_1_, AuAgPd trimetallic nanoparticles; AgNPs@Ti_3_C_2_ NSs, Ag nanoparticle-decorated Ti_3_C_2_ nanosheets; IrO_2_/rGO nanocomposites, ultrafine IrO_2_ nanoparticles on reduced graphene oxide nanosheets.

**Table 2:** Comparison of the kinetic parameters of different materials with hydrolase-like activity using 4-MUP as the substrate.

Catalyst	Substance	*K*_m_ (μM)	*V*_max_ (10^−8^ M/s)	Ref.
ALP	4-MUP	1.13	0.19	[51]
CeO_2_ NPs		16.59	2.76	[51]
ZrO_2_ NPs		14.70	0.53	[51]
Ce(IV)/QDs		4.55	1.34	[52]
MIP-202(Zr)		10.27	0.04	[53]
Ce-UiO-66-NO_2_		8.15	0.94	[54]
CeO_2_ NFs		27.93	36.63	This work

Abbreviations: ALP, alkaline phosphatase; CeO_2_ NPs, cerium dioxide nanoparticles; ZrO_2_ NPs, zirconium oxide nanoparticles; Ce(IV)/QDs, Ce(III) ion-modified fluorescent quantum dots oxidized by reactive oxygen species to form nanozyme; MIP-202(Zr), α-amino acid–zirconium polymer metal–organic framework; Ce-UiO-66-NO_2_, Ce doped into Zr(IV)-based metal–organic framework.

**Table 3:** Comparison with previously reported methods for the recognition of L/D-Arg.

Detection System	Analyte	Detection Range (μM)	LOD (μM)	Recognition Difference (ef)	Ref.
UiO-66-NH_2_	Arg	0–645	21.50	-	[55]
dual-emission CDs	Arg	27–107	9.16	-	[56]
TBAPy probe	Arg	0–200	2.30	-	[57]
MPA-QDs	L/D-Arg	100–6000, 12,000–40,000 /500–2500, 9000–40,000	8.00/15.00	-	[58]
aptamer AuNps	L/D-Arg	0.025–0.40	0.0018	-	[59]
β-CD@L-MnOx	L/D-Arg	0–290	11.91/19.56	-	[60]
L-ZnO-coated QCM	L/D-Arg	0–10,000	510.00/850.00	1.54	[61]
fluorescent probe (*R*)-3	L/D-Arg	40–400	0.12/0.060	1.80	[62]
CeO_2_ NFs	L/D-Arg	770–940	26.00/79.00	2.48	This work

Abbreviations: UiO-66-NH_2_, amino group-functionalized Zr(IV)-based metal–organic framework; CDs, carbon dots; TBAPy, 1,3,6,8-Tetrakis (p-benzoic acid) pyrene; MPA-QDs, mercaptopropionic acid-capped CdTe quantum dots; aptamer AuNps, gold nanoparticles coated with aptamers; β-CD@L-MnOx, β-Cyclodextrin integrated into mixed-valence L-MnO_x_ species (Mn^2+^/Mn^3+^/Mn^4+^); L-ZnO-coated QCM, chiral zinc oxide-functionalized quartz crystal microbalance sensor.

## Data Availability

The original contributions presented in this study are included in the article. Further inquiries can be directed to the corresponding authors.

## Supplementary Materials

The following supporting information can be downloaded at: https://www.mdpi.com/article/10.3390/molecules31122003/s1, Text S1: Reagents and chemicals; Text S2: Apparatus and measurements; Text S3: DFT calculations; Figure S1: SEM images of the as-synthesized CeO_2_ NFs (165 °C for 2 h and 0.45 g PVP) with different concentrations of Ce(III): 1.0 M (A), 1.2 M (B),1.4 M (C), and 1.6 M (D). FT-IR spectra (E) and XRD patterns (F) of CeO_2_ NFs synthesized with different Ce(III) concentrations. The effect of Ce(III) concentrations on the chiral recognition of Arg resulting in a fluorescence intensity (G) and fluorescence intensity ratio (H) change; Figure S2: SEM image of the as-synthesized CeO_2_ NFs with Ce(III) (1.4 M) and PVP (0.45 g) at 165 °C for 1 h (A), 2 h (B), 3 h (C), and 4 h (D). FT-IR spectra (E) and XRD patterns (F) of CeO_2_ NFs synthesized for different synthesis times. The effect of synthesis time on the chiral recognition of Arg resulting in a fluorescence intensity (G) and fluorescence intensity ratio (H) change; Figure S3: SEM images of the as-synthesized CeO_2_ NFs at 165 °C for 3 h with 1.4 M of cerium with different amounts of PVP: 0.3 g (A), 0.55 g (B), 0.65 g (C), and 0.75 g (D). FT-IR spectra (E) and XRD patterns (F) of CeO_2_ NFs synthesized with different PVP amounts. The effect of PVP amount on the chiral recognition of Arg by a fluorescence analysis (G and H); Figure S4: The effect of dilution multiple of CeO_2_ NFs (A), buffer pH (B), H_2_O_2_ (C) and OPD (D) concentrations, reaction temperature (E), and time (F) on the chiral recognition of Arg based on the POD-like activity of CeO_2_ NFs; Figure S5: The effect of buffer pH (A), dilution multiple of CeO_2_ NFs (B), reaction temperature (C), concentration of 4-MUP (D), and reaction time (E) on the detection of *p*-NP and ALP based on the hydrolase-like activity of CeO_2_ NFs; Figure S6: Catalytic stability of different batches of CeO_2_ NFs for peroxidase-like (A) and hydrolase-like (B) activities. Error bars represent standard deviation (SD) of three independent measurements (*n* = 3); Table S1: Detection of L-Arg in rabbit plasma; Table S2: Detection of *p*-NP in water samples; Table S3: Detection of ALP in rabbit plasma.
