# Supplementary material for: Dual-Functional CeO2 Nanozyme-Based Fluorescent Sensing Platform for Chiral Recognition of Arginine and “On-Off-On” Detection of p-Nitrophenol and Alkaline Phosphatase
Source: Molecules. 2026 Jun 8;31(12):2003. doi: 10.3390/molecules31122003 (PMC13304681; doi:10.3390/molecules31122003)
Supplement: Supplementary file 1 [file molecules-31-02003-s001.zip › molecules-4330918-supplementary.pdf]

Supplementary Materials

Dual-functional CeO_2 nanozyme based fluorescent sensing platform for chiral recognition of arginine and “on-off-on” detection of *p*-nitrophenol and alkaline phosphatase

Hui-Ling Chen, Jing-Jing Dai, Hua Chen, Guo-Ying Chen*, Feng-Qing Yang*

School of Chemistry and Chemical Engineering, Chongqing University, Chongqing 401331, China.

*Corresponding Authors:

20221801017@stu.cqu.edu.cn (G.-Y.C), fengqingyang@cqu.edu.cn (F.-Q.Y).

Supplementary Method

Text S1: Reagents and Chemicals

The $Ce(NO_3)_3·6H_2O$, $C_2H_6O_2$ (98%), D-arginine (>98%), 4-methylumbelliferyl phosphate, and D-glutamic acid (>98%) were purchased from Macklin Biochemical Technology Co., Ltd. (Shanghai, China). *o*-Phenylenediamine (99%), superoxide dismutase, gallic acid (98%), L-cysteine (>99%), D-aspartic acid (>99%), D-methionine (>99%), D-alanine (>98%), D-proline (>99%), D-threonine (>99%), and D-valine (>98%) were purchased from Adamas-beta Co., Ltd. (Shanghai, China). KI, Co $(NO_3)_2$, *p*-nitrophenol, and L-glutamic acid (99%) were purchased from Shanghai Aladdin Bio-chemical Technology Co., Ltd. (Shanghai, China). Polyvinylpyrrolidone (AR), alkaline phosphatase (60 U/μL), L-aspartic acid (99%), and the rabbit plasma (a biological product with sodium citrate as anticoagulant) were purchased from Shanghai YuanYe Biological Technology Co., Ltd. (Shanghai, China). NaCl, KCl, 30% H_2O_2, and $AgNO_3$ were purchased from Chengdu Chron Chemicals Co., Ltd. (Chengdu, China). CH_3O and $C_4H_{10}O$ were purchased from Chengdu Kelong Chemical Reagent Factory (Chengdu, China). L-lysine (BR), L-arginine (BR), L-tyrosine (AR), and L-histidine (>99.5%) were purchased from Chengdu Huaxia Chemical Reagent Co., Ltd. (Chengdu, China). D-cysteine (99%), D-tyrosine (98%), L-serine (99%), D-asparagine (99%), D-leucine (99%), and L-leucine (99%) were purchased from Heowns Biochem Technologies (Tianjin, China). D-glutamine (99%), D-isoleucine (98%), L-asparagine (99%), and L-threonine (99%) were purchased from Beijing Mreda Technology Co., Ltd. (Beijing, China). L-isoleucine (99%), D-histidine (>98%), D-tryptophan (>99%), and L-valine (99%) were purchased from Shanghai Mairel Chemical Technology Co., Ltd. (Shanghai, China). D-lysine (>98%) was purchased from Bide Pharmatech Co., Ltd. (Shanghai, China). L-methionine (98%)

and D-serine (>98%) were purchased from Energy Chemical (Shanghai, China). L-proline (99%) was purchased from Beijing Boaotuoda Technology Co., Ltd. (Beijing, China). L-alanine (BR) was purchased from Shanghai Yien Chemical Technology Co., Ltd. (Shanghai, China). L-tryptophan (AR) was purchased from Beijing Dingguo Biotechnology Co., Ltd. (Beijing, China). L-glutamine (≥98.5%) was purchased from Beijing Notles Biotechnology Co., Ltd. (Beijing, China). L-phenylalanine (99.5%) was purchased from Sangon Biotech Co., Ltd. (Shanghai, China). D-phenylalanine (BR) was purchased from Sinopharm Chemical Reagent Co., Ltd. (Beijing, China). All the samples were deposited at the Pharmaceutical Engineering Laboratory in the School of Chemistry and Chemical Engineering, Chongqing University, Chongqing, China.

Text S2: Apparatus and Measurements

The field-emission scanning electron microscopy (SEM) (JSM-7600F, JEOL Ltd., Tokyo, Japan) was used to characterize the synthesized materials. The Fourier-transform infrared (FT-IR) spectra was recorded using a Nicolet iS50 (Thermo Scientific Inc., USA). The sample's X-ray diffraction (XRD) patterns were acquired through an X′ pert Powder diffractometer (Malvern Panalytical Ltd., Netherlands). Transmission electron microscopy (TEM) images and element distribution analysis were recorded using a JEM 2100 electron microscope (JEOL Ltd. Tokyo, Japan) working at 200 kV, which is equipped with energy dispersive X-ray spectrometer (EDX). Thermogravimetric analysis (TGA) was carried out on the Mettler TGA/DSC1/1600LF (Mettler-Toledo AG, Analytical, Switzerland). X-ray photoelectron spectrometry (XPS) was recorded on a PHI5000 Versaprobe system using monochromatic Al Ka radiation (1486.6 eV), and the obtained binding energies were referenced to the C 1s line set at 284.8 eV (Thermo Fisher Scientific Ltd, UK). Zeta potential was measured using a NanoBrook Omni

instrument (Brookhaven Instruments Corporation, UK). The UV-Vis spectra were obtained on a UV-5500 PC spectrophotometer (Shanghai, China). Fluorescence spectra was measured using an F-7100 fluorescence spectrophotometer (Hitachi, Tokyo, Japan). A tabletop low-speed centrifuge (L420, Hunan Xiangyi Laboratory Instrument Development Co., Ltd., Hunan, China) was employed for sample separation. Temperature control was achieved using a drying oven (DHG-9146A, Longyue Instrument Equipment, Shanghai, China). A FE28 pH meter (Mettler-Toledo Instruments, Shanghai, China) was used for measuring the pH of solutions. The ultrapure water (18.25 MΩ·cm) used throughout this study was purified by a water purification system (ATSelem 1820A, Antesheng Environmental Protection Equipment, Chongqing, China). The ultrasonic cleaner was purchased from Kunshan Jielimei Ultrasonic Instrument Co., Ltd. (Jiangsu, China).

Text S3: DFT calculations

All calculations were performed using the Gaussian 09 software suite, with structural analysis and visualization carried out in GaussView 5.0. Geometry optimizations employed the B3LYP functional and a 6-311++G(d,p) basis set, followed by vibrational frequency analyses at the same level to confirm the absence of imaginary frequencies and ensure that the structures correspond to true minima on the potential energy surface. Subsequently, single-point energy calculations were carried out on the optimized structures using the B3LYP/6-311++G(d,p) (ignoring the effect of the solvent). Geometric structure optimization was performed separately for the three individual molecules (PVP, L-Arg, and D-Arg) and for the complexes of PVP with L-Arg and D-Arg. The binding energy (E) between PVP and L/D-Arg was calculated according to the equation: $E = E_{complex} - E_{PVP} - E_{L/D\text{-}Arg}$, where E_{PVP} and $E_{L/D\text{-}Arg}$ represent the energies

of the isolated PVP and the respective L/D-Arg molecule, respectively, and $E_{complex}$ is the energy of the optimized complex.

Supplementary Figures

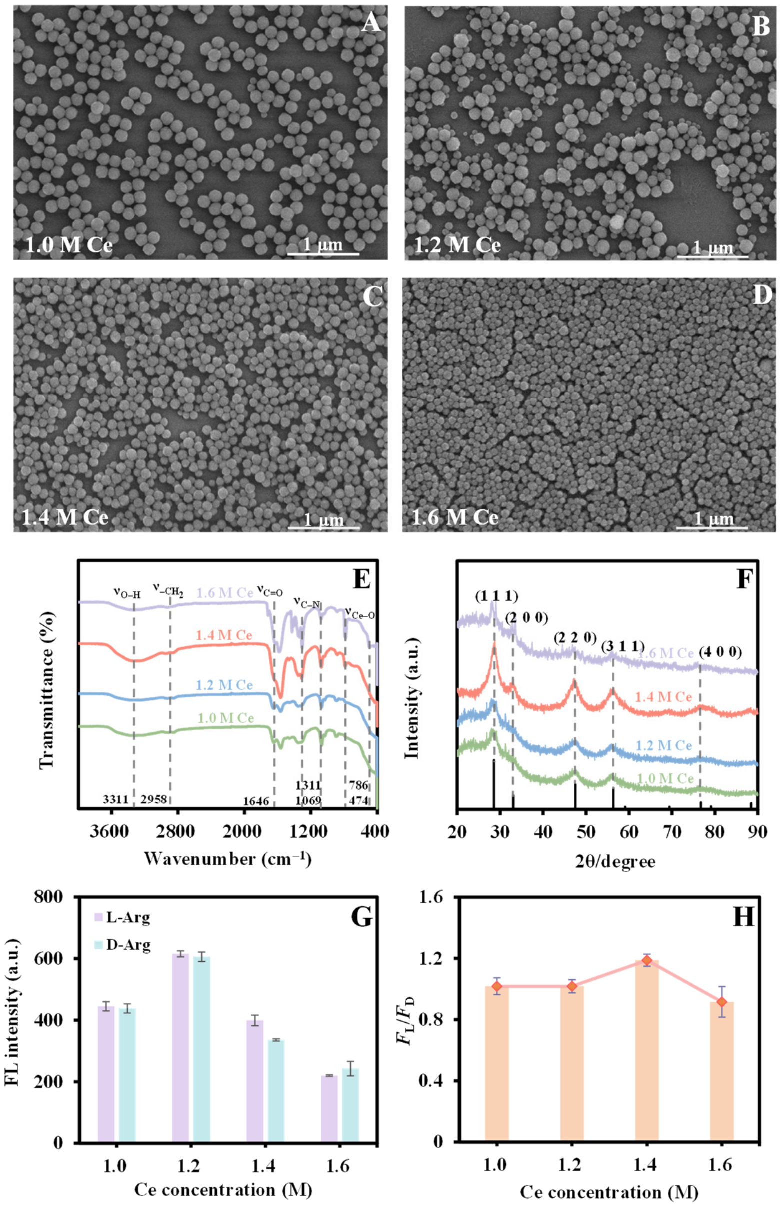

Figure S1. SEM images of the as-synthesized CeO_2 NFs (165 °C for 2 h and 0.45 g PVP) with different concentrations of Ce(III) 1.0 M (A) ,1.2 M (B),1.4 M (C), and 1.6 M (D). FT-IR spectra (E) and XRD patterns (F) of CeO_2 NFs synthesized with different Ce(III) concentrations. The effect of Ce(III) concentrations on the chiral recognition of Arg resulting in the fluorescence intensity (G) and fluorescence intensity ratio (H) change.

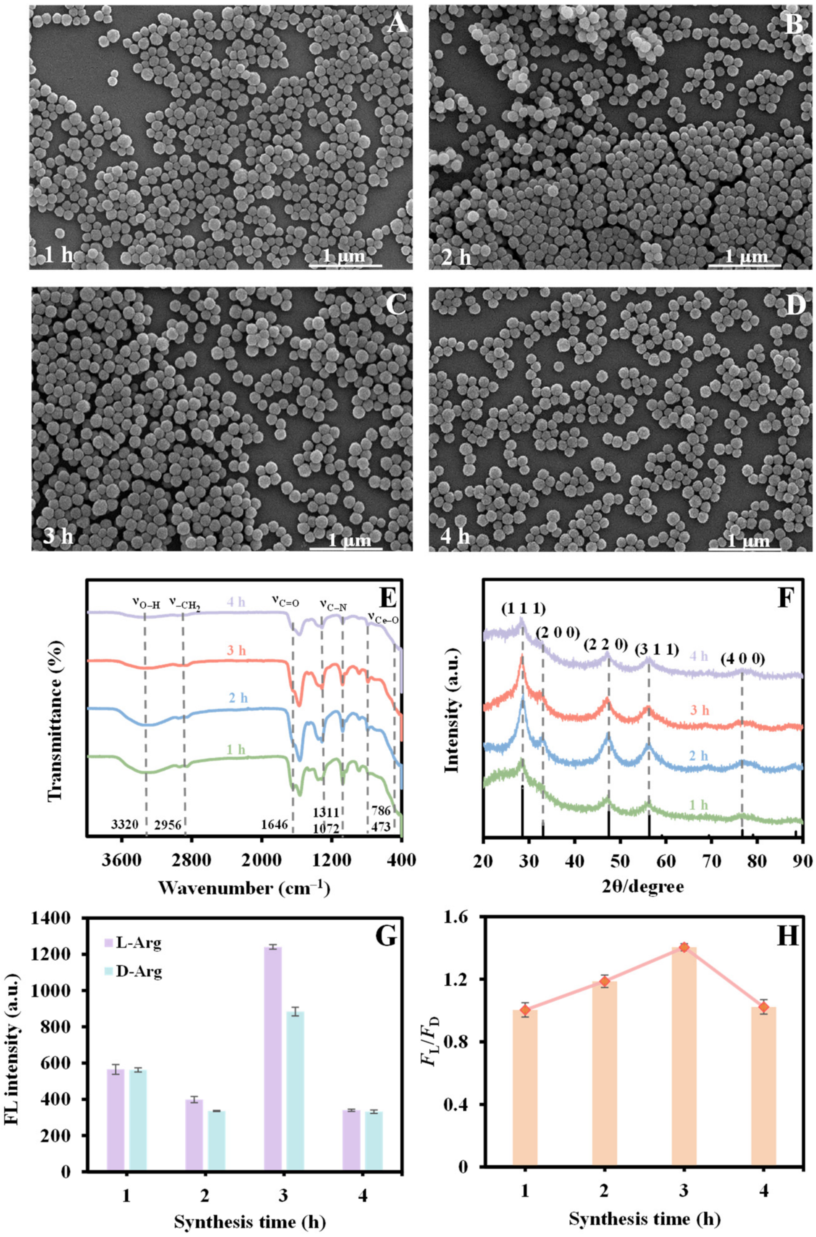

Figure S2. SEM image of the as-synthesized CeO_2 NFs with Ce(III) (1.4 M) and PVP (0.45 g) at 165 °C for 1 h (A), 2 h (B), 3 h (C), and 4 h (D). FT-IR spectra (E) and XRD patterns (F) of CeO_2 NFs synthesized with different synthesis time. The effect of synthesis time on the chiral recognition of Arg resulting in the fluorescence intensity (G) and fluorescence intensity ratio (H) change.

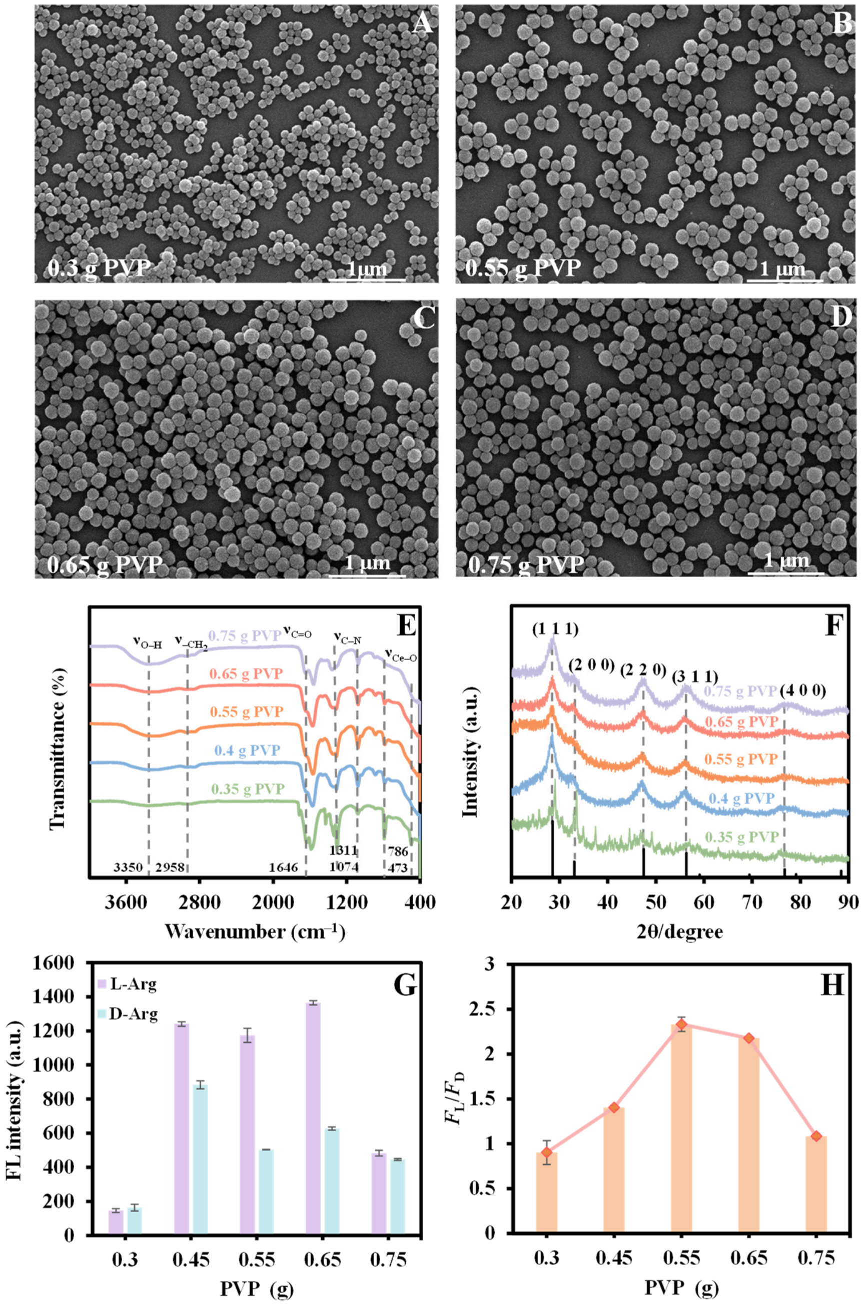

Figure S3. SEM images of the as-synthesized CeO_2 NFs at 165 °C for 3 h and 1.4 M of cerium with different amount of PVP: 0.3 g (A), 0.55 g (B), 0.65 g (C), and 0.75 g (D). FT-IR spectra (E) and XRD patterns (F) of CeO_2 NFs synthesized with different PVP amount. The effect of PVP amount on the chiral recognition of Arg by fluorescence analysis (G and H).

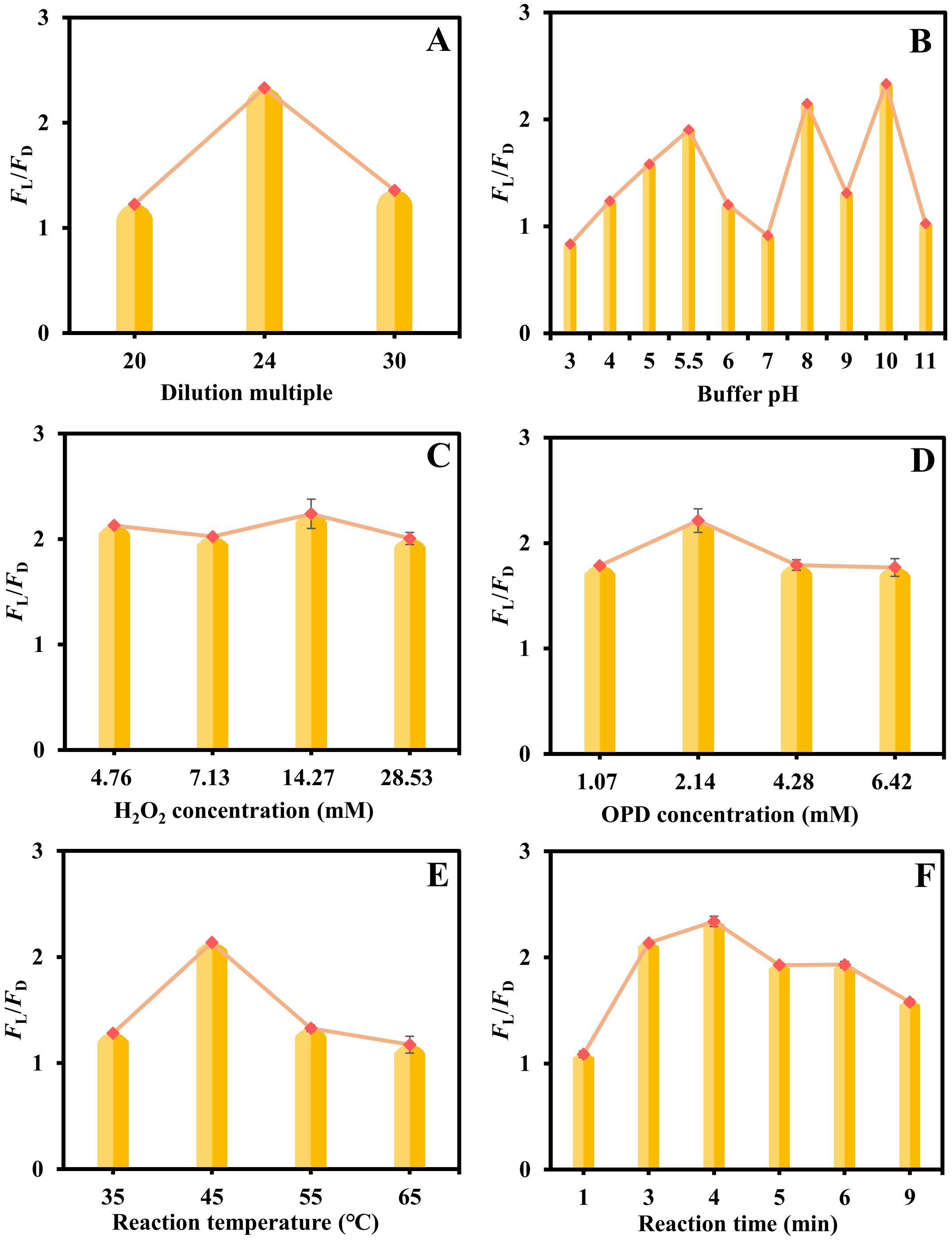

Figure S4. The effect of dilution multiple of CeO_2 NFs (A), buffer pH (B), H_2O_2 (C) and OPD (D) concentrations, reaction temperature (E), and time(F) on the chiral recognition of Arg based on the POD-like activity of CeO_2 NFs.

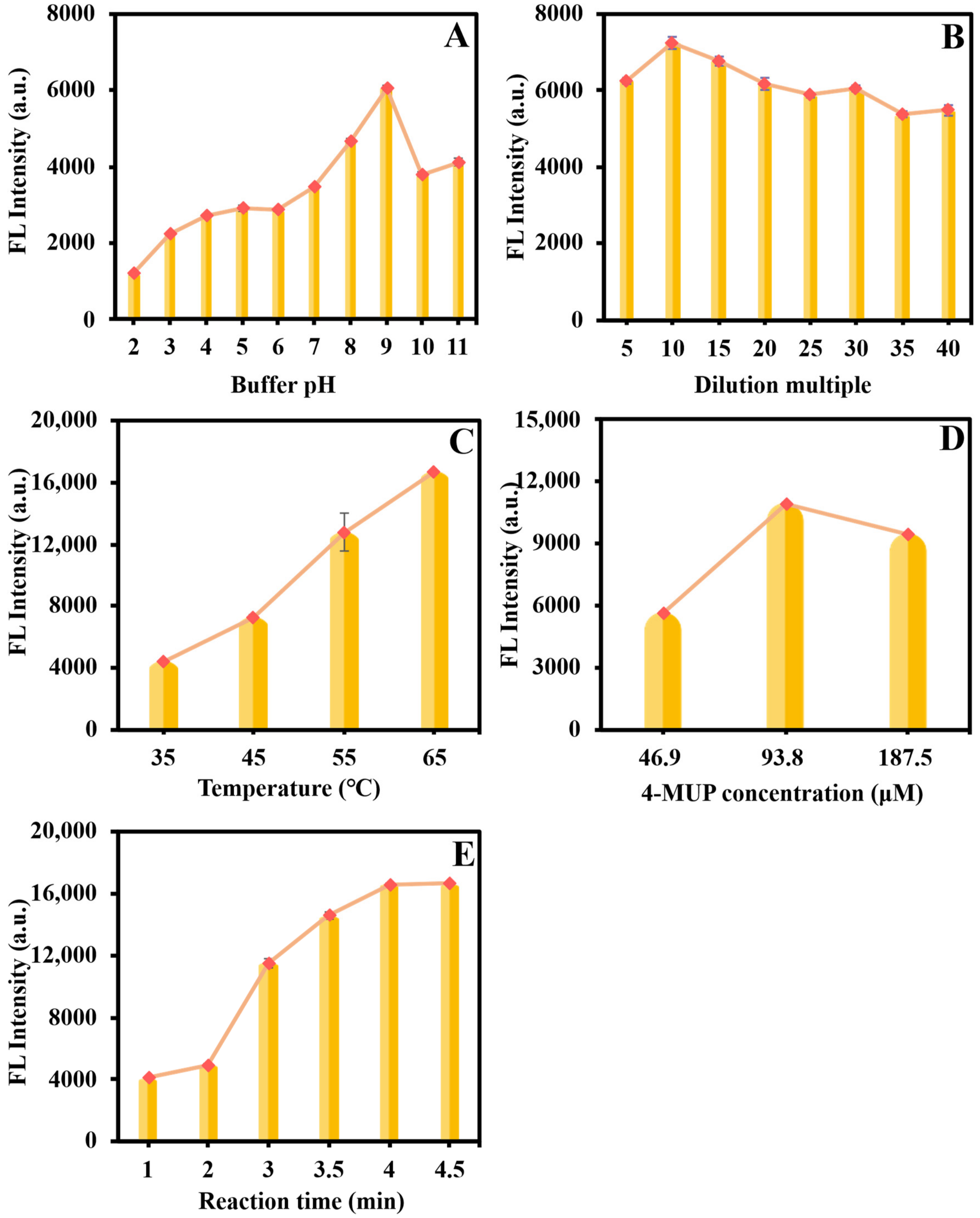

Figure S5. The effect of buffer pH (A), dilution multiple of CeO_2 NFs (B), reaction temperature (C), concentration of 4-MUP (D), and reaction time(E) on the detection of *p*-NP and ALP based on the hydrolase-like activity of CeO_2 NFs.

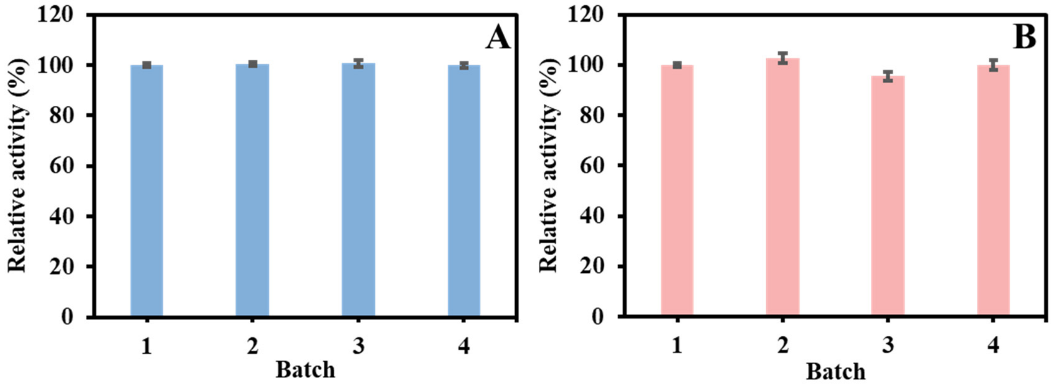

Figure S6. Different batches catalytic stability of CeO_2 NFs for peroxidase-like (A) and hydrolase-like (B) activities. Error bars represent the standard deviation (SD) of three independent measurements ($n = 3$).

Supplementary Tables

Table S1. Detection of L-Arg in rabbit plasma.

Sample	Added (mM)	Found (mM)	Recovery (%)	RSD (%, n = 3)
Rabbit plasma	0.81	0.77	94.7	2.1
	0.86	0.89	104.0	0.6
	0.90	0.97	108.1	0.2

Table S2. Detection of *p*-NP in water samples.

Sample	Added (mM)	Found (mM)	Recovery (%)	RSD (%, n = 3)
Yun Lake water	0.22	0.20	89.3	2.2
	0.15	0.15	98.5	3.0
	0.075	0.068	91.1	3.1
Jin Lake water	0.22	0.20	89.1	1.9
	0.15	0.15	101.2	3.8
	0.075	0.078	103.6	3.4
Tap water	0.22	0.20	90.5	1.1
	0.15	0.15	101.6	2.5
	0.075	0.071	94.9	4.5

Table S3. Detection of ALP in rabbit plasma.

Sample	Added (U/mL)	Found (U/mL)	Recovery (%)	RSD (%, n = 3)
Rabbit plasma	0.60	0.56	92.8	4.3
	1.00	0.97	97.5	3.9
	1.50	1.19	80.6	2.2
